# Regulation of Leptin Methylation Not via Apoptosis by Melatonin in the Rescue of Chronic Programming Liver Steatosis

**DOI:** 10.3390/ijms19113565

**Published:** 2018-11-12

**Authors:** Ching-Chou Tsai, Yu-Ju Lin, Hong-Ren Yu, Jiunn-Ming Sheen, I-Chun Lin, Yun-Ju Lai, You-Lin Tain, Li-Tung Huang, Mao-Meng Tiao

**Affiliations:** 1 Department of Obstetrics and Gynecology, Kaohsiung Chang Gung Memorial Hospital and Chang Gung University, Kaohsiung 83301, Taiwan; nickcctsai@yahoo.com.tw (C.-C.T.); lyu015ster@gmail.com (Y.-J.L.); lusionbear@cgmh.org.tw (Y.-J.L.); 2 Graduate Institute of Clinical Medicine, Kaohsiung Medical University, Kaohsiung 80708, Taiwan; 3 Department of Pediatrics, Kaohsiung Chang Gung Memorial Hospital and Chang Gung University, Kaohsiung 83301, Taiwan; yuu2004taiwan@yahoo.com.tw (H.-R.Y.); ray.sheen@gmail.com (J.-M.S.); uc22@cgmh.org.tw (I.-C.L.); tainyl@hotmail.com (Y.-L.T.); litung.huang@gmail.com (L.-T.H.)

**Keywords:** melatonin, steatosis, leptin, prenatal dexamethasone, programming, liver

## Abstract

We examined the mechanisms of chronic liver steatosis after prenatal dexamethasone exposure and whether melatonin rescues adult offspring with liver steatosis. Melatonin rescued prenatal dexamethasone-exposed livers with steatosis in young rats. Sprague-Dawley rats pregnant at gestational day 14–21 were administered with intraperitoneal dexamethasone (DEX) or prenatal dexamethasone and melatonin between gestational day 14 and postnatal day ~120 (DEX+MEL). Chronic programming effects in the liver were assessed at day ~120. Liver steatosis increased in the DEX compared with that in the vehicle group and decreased in the DEX+MEL group (*p* < 0.05), with no changes in cellular apoptosis. Expression of leptin and its receptor decreased in the DEX (*p* < 0.05) and increased in the DEX+MEL group (*p* < 0.05), as revealed by RT-PCR and Western blotting. Tumor necrosis factor alpha (TNF-α) and interleukin (IL)-6 expression increased in the DEX group compared with that in the vehicle group and decreased in the DEX+MEL group (*p* < 0.05). Liver DNA methyltransferase activity and leptin methylation increased in the DEX group (*p* < 0.05) and decreased in the DEX+MEL group (*p* < 0.05), with no changes in HDAC activity. Thus, prenatal dexamethasone induces liver steatosis at ~120 days via altered leptin expression and liver inflammation without leptin resistance. Melatonin reverses leptin methylation and expression and decreases inflammation and chronic liver steatosis not via apoptosis or histone deacetylation (HDAC).

## 1. Introduction

Clinically, 7% of pregnant women are at risk of preterm delivery. In these cases, the women are routinely treated with glucocorticoids in an attempt to improve neonatal outcome [1,2] by accelerating fetal lung maturation [3,4]. However, prenatal overexposure to glucocorticoids in humans [5] and rats has been shown to increase susceptibility to fatty liver disease with liver steatosis [2,6], and has negative implications on the health of the resulting offspring, which can persist into adulthood [5,6]. Epigenetic regulation during prenatal life has thus been shown to result in fatty liver disease in adulthood [7]. This may be the result of depot-specific-programmed alterations in fat metabolism transcriptions in adipose tissue [8,9,10].

The adipocyte hormone leptin is a critical modulator of both acute and long-term metabolic health and controls energy expenditure [11]. Leptin deficiency causes morbid obesity and fatty liver disease in mice and humans [12,13]. Leptin resistance has been previously reported in relation to studies on liver disease [14]. Melatonin is the main product of the pineal gland and has wide-ranging effects on inflammatory cells [15,16] and the serum total cholesterol with triglycerides [17]. We have previously reported that melatonin can rescue liver steatosis in seven-day-old rats via leptin methylation and cellular apoptosis [2]. However, a strategy is still needed for the rescue of chronic liver steatosis.

The implications of prenatal insults on the risk of developing disease in adulthood provides a basis for the study of how epigenetic manipulation may lead to chronic liver steatosis disease [10]. However, the mechanism and management of this long-term programming of prenatal dexamethasone exposure in the liver is not yet fully understood. We hypothesized that the chronic programming effect of prenatal dexamethasone on liver steatosis involves leptin epigenetic modifications with liver inflammatory changes, wherein melatonin may play a protective role in the progression of chronic liver steatosis.

## 2. Results

### 2.1. Liver Steatosis

**Figure 1:**
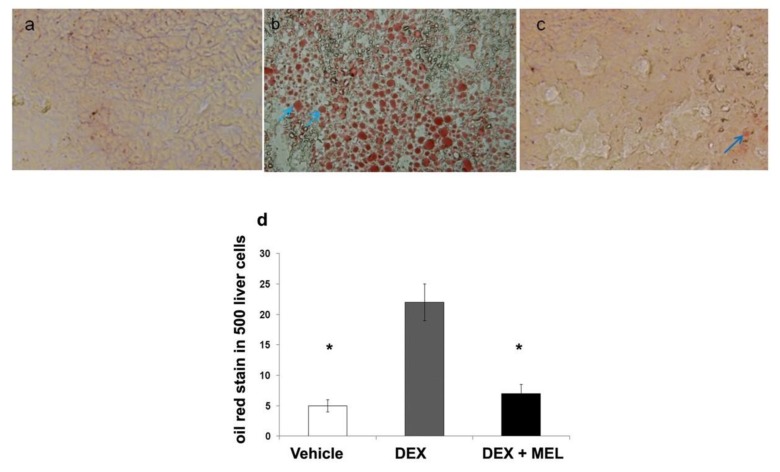
Liver steatosis. Oil red staining showed overexpression in the prenatal dexamethasone group (DEX) compared with that in the vehicle group. This was found to return to the control value in the prenatal dexamethasone and melatonin treatment group (DEX+MEL). (**a**) Oil red staining in the vehicle group. (**b**) The increased liver cell oil red staining in the DEX group. (**c**) The melatonin treatment decreased the number of oil red stained liver cells induced by dexamethasone at ~120 days (original magnification ×400, blue arrows: positive hepatocytes). (**d**) Semi-quantification of the oil red stained cells. All the results represent the standard error of means of six animals, * *p* < 0.05 when comparing with the DEX group. The letters represent different groups. DEX, prenatal dexamethasone; DEX+MEL, melatonin treatment after prenatal dexamethasone.

Liver steatosis in ~120-day-old rats was studied by oil red staining and was found to be overexpressed in the prenatal dexamethasone group (DEX) compared with that in the vehicle group and returned to the control value in the melatonin treatment group (DEX+MEL) (Figure 1a–d). The liver weight was significantly increased in the prenatal dexamethasone (DEX) for the liver steatosis model. The body weight and triglyceride levels did not increase in the prenatal dexamethasone group (DEX), but decreased in the melatonin treatment group (DEX+MEL) (Table 1).

### 2.2. Leptin Expression and Methylation

**Figure 2:**
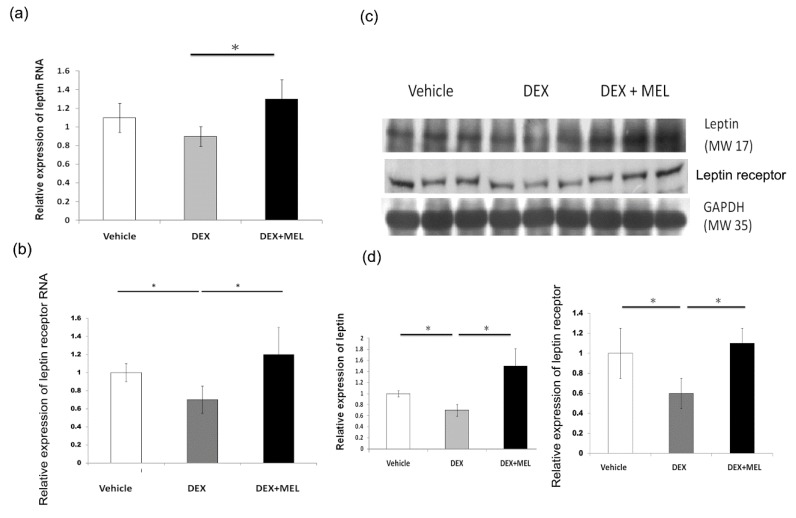
Leptin expression and methylation. (**a**,**b**) Real-time PCR study. The levels of leptin and leptin receptor expression decreased in the DEX group compared with that in the vehicle group and increased in the DEX+MEL group. (**c**) Western blotting showed decreased leptin and leptin receptor expression in the DEX group compared with that in the vehicle group. This expression was restored in the DEX+MEL group. (**d**) Semi-quantification of Western blot expression of leptin and leptin receptor. All results represent the mean ± standard error of six animals; * *p* < 0.05 when comparing DEX with the vehicle group or DEX+MEL with the DEX group. The letters represent different groups. DEX, prenatal dexamethasone; DEX+MEL, melatonin treatment after prenatal dexamethasone.

Real-time PCR showed a decreased expression of leptin and its receptor in the DEX group compared with that in the vehicle group, which was recovered in the DEX+MEL group. (Figure 2a,b) Western blotting analysis showed decreased leptin and leptin receptor expression in the DEX group compared with that in the vehicle group. Leptin and leptin receptor expression was found to be restored in the DEX+MEL group. (Figure 2c,d). No leptin resistance was found in the DEX group, with lower leptin and leptin RNA levels but no increased cholesterol levels. The PEPCK and IGF1 protein expression levels were not significantly different among the groups according to the Western blot (data not shown).

### 2.3. Expression of Leptin Methylation

**Figure 3:**
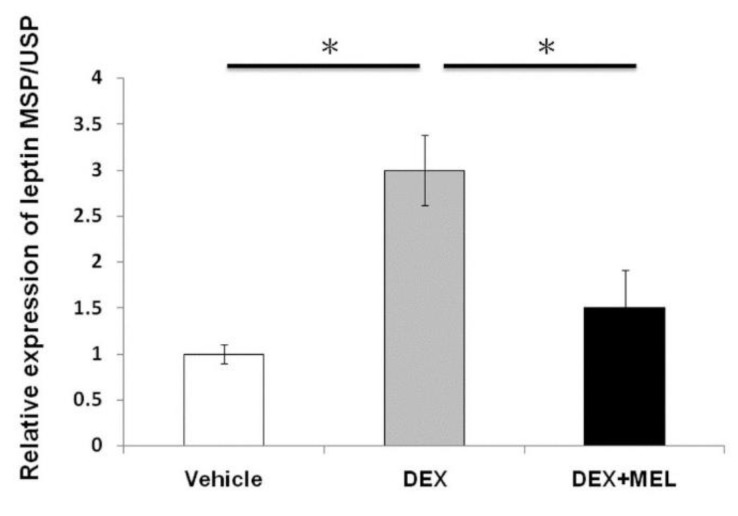
Expression of leptin methylation. Leptin methylation-specific PCR/unmethylation-specific PCR (MSP/USP) showed that the DEX group had increased levels of leptin methylation, with those levels decreasing after melatonin treatment. All the results represent the mean ± standard error of six animals, * *p* < 0.05 when comparing the DEX group with vehicle group or the DEX+MEL group with the DEX group. The letters represent different groups. DEX, prenatal dexamethasone; DEX+MEL, melatonin treatment after prenatal dexamethasone.

Total DNA methylation was studied by methylation-specific PCR/unmethylation-specific PCR (MSP/USP). The results showed that the DEX group had an increased level of leptin methylation and a decreased level in the DEX+MEL group (Figure 3).

### 2.4. Histone Deacetylation (HDAC) and DNA Methyltransferase (DNMT) Activity in Liver

**Figure 4:**
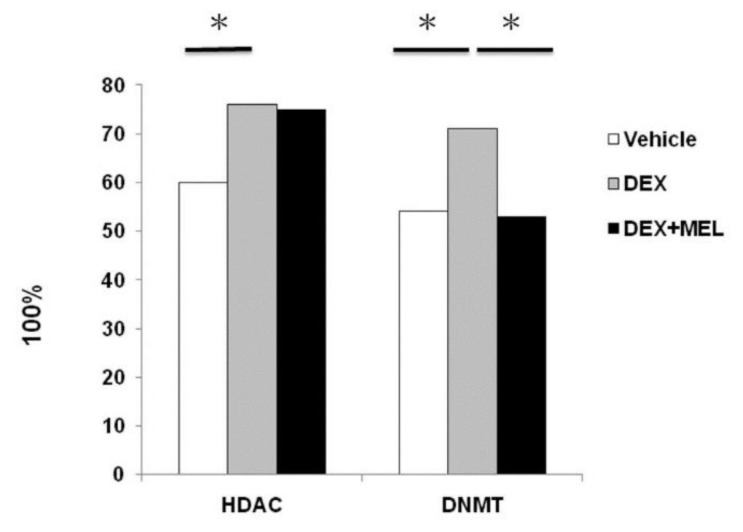
Histone deacetylation (HDAC) and DNA methyltransferase (DNMT) activity in the liver. The histone modifications in fatty liver were studied via HDAC activity and leptin DNMT activity. Our data showed that DNMT increased in the DEX group compared with that in the vehicle group and decreased in the DEX+MEL group. HDAC activity increased in the DEX group compared with that in the vehicle group, but did not decrease in the DEX+MEL group. All the results represent the mean ± standard error of six animals; * *p* < 0.05 when comparing the DEX group with the vehicle group or the DEX+MEL group with the DEX group. The letters represent different groups. DEX, prenatal dexamethasone; DEX+MEL, melatonin treatment after prenatal dexamethasone.

The histone modifications in the fatty liver were studied by measuring HDAC activity and leptin DNMT activity. Our data showed that DNMT increased in the DEX group compared with that in the vehicle group and decreased in the DEX+MEL group. HDAC increased in the DEX group compared with that in the vehicle group, but was not found to decrease in the DEX+MEL group (Figure 4).

### 2.5. Inflammation and Apoptosis Studies

**Figure 5:**
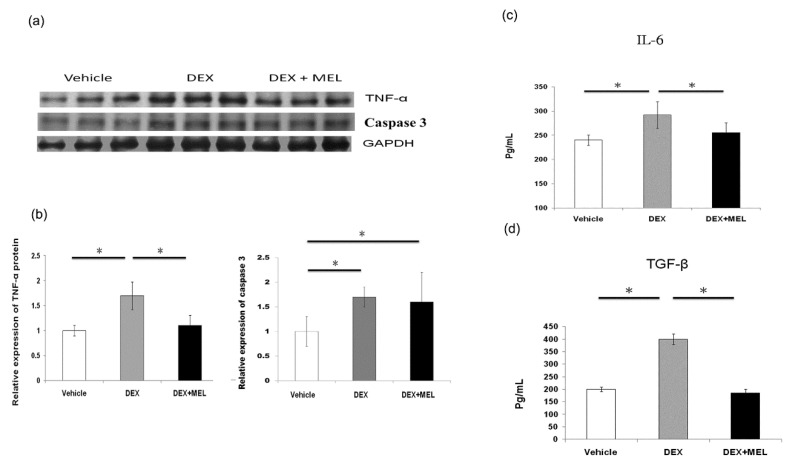
Inflammatory and apoptosis study. Increased tumor necrosis factor alpha (TNF-α) and caspase 3 protein expression was seen in the DEX group compared with that in the vehicle group and TNF-α, not caspase 3, decreased in the DEX+MEL group (**a**,**b**). Enzyme-linked immunosorbent assay (ELISA) showed interleukin (IL)-6 and transforming growth factor beta (TGF-β) levels were increased in the DEX group compared with that in the vehicle group and decreased in the DEX+MEL group (**c**,**d**). All the results represent the standard error of mean (SEM) of six animals; * *p* < 0.05 when comparing with the DEX group. The letters above each represented different groups with DEX representing the prenatal dexamethasone; DEX+MEL, melatonin treatment after prenatal dexamethasone.

**Figure 6:**
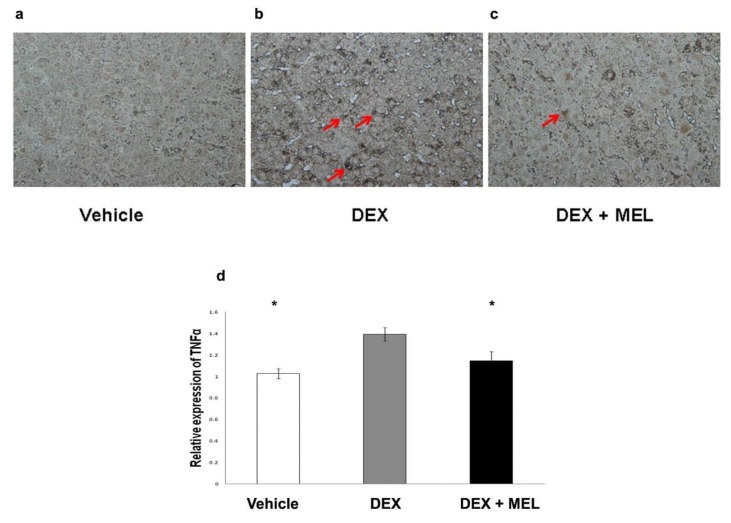
The TNF-α immunohistochemistry showed (**a**–**c**) increased staining in the prenatal steroid compared with the control group and decreased in the melatonin group (original magnification ×400, red arrows: positive hepatocytes). (**d**) Semi-quantification of the TNF-α immunohistochemistry staining. All the results represent the mean ± standard error of six animals; * *p* < 0.05 when comparing with DEX group. The letters above each represented different groups with DEX representing the prenatal dexamethasone; DEX+MEL, melatonin treatment after prenatal dexamethasone.

**Figure 7:**
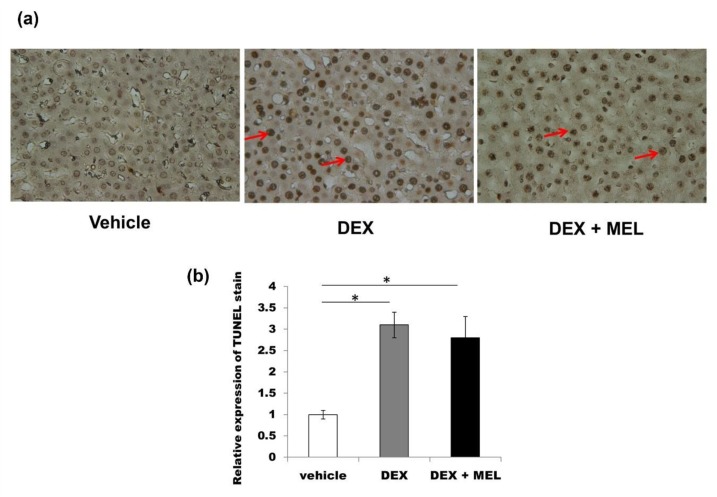
The extent of TdT-mediated dUTP biotin nick end labeling (TUNEL) staining was assessed for whether apoptosis was involved in this liver damage. (**a**) It was overexpressed in the DEX group compared with the vehicle group, but not decreased in the DEX+MEL group. (**b**) Semi-quantification of the TUNEL stained cells. All the results represent standard error of mean (SEM) of six animals; * *p* < 0.05. The letters above each represented different groups with DEX representing the prenatal dexamethasone; DEX+MEL, melatonin treatment after prenatal dexamethasone (original magnification ×400, red arrows: apoptosis).

Tumor necrosis factor alpha (TNF-α) protein expression was found to be increased in the DEX group compared with that in the vehicle group and decreased to control values in the DEX+MEL group (Figure 5a,b). The caspase 3 protein expression was found to be increased in the DEX group compared with that in the vehicle group, but not significantly decreased in the DEX+MEL group (Figure 5a,b). Enzyme-linked immunosorbent assay (ELISA) showed that the levels of interleukin (IL)-6 and transforming growth factor beta (TGF-β) expression were increased in the DEX group compared with those in the vehicle group and decreased in the DEX+MEL group (Figure 5c,d). Increased TNF-α staining was found in the DEX group when compared with the vehicle group and decreased to control values in the DEX+MEL group (Figure 6a–d). To assess whether apoptosis is involved in liver damage as in the acute stage [2], the activation of the apoptotic machinery was measured using activated TdT-mediated dUTP biotin nick end labeling (TUNEL) staining. TUNEL staining revealed a significantly greater proportion of apoptotic cells in the DEX group than in the other two groups (Figure 7). Following melantonin therapy in the DEX+MEL group, the degree of TUNEL staining was decreased in comparison with that in the DEX group. Our data showed that apoptosis levels were not significantly different between the DEX and DEX+MEL groups, which was not correlated with the levels of the acute stage [2].

## 3. Discussion

The present study showed that melatonin can rescue chronic liver steatosis in prenatal dexamethasone-exposed adult offspring. The mechanisms for chronic liver steatosis programming were found to function via leptin methylation and liver inflammation, but not leptin resistance. Melatonin decreased chronic liver steatosis by lowering leptin methylation, restoring leptin expression, and reducing inflammation. However, this is found to not occur via the cellular apoptosis or HDAC pathways.

Steatosis in nonalcoholic fatty liver disease (NAFLD) is the most common cause of chronic liver disease in adults and may lead to developing liver fibrosis with cirrhosis [18,19,20]. The modifications of the human genome through epigenetic processes during prenatal life are more likely to predispose an individual to chronic disease in adult life [3,7,8,9,10]. Many studies have shown that prenatal glucocorticoid overexposure in rats increases liver lipid accumulation with steatosis [2,5,6,8], which may be the result of programmed alterations in the fat metabolism [2,8]. In this study, we showed that chronic liver steatosis can be caused by prenatal glucocorticoid overexposure.

Leptin deficiency causes morbid obesity with fatty liver in mice and humans [12,13,21], and can lead to chronic liver disease [22]. This supports the possibility of treating obesity via the leptin pathway [21], which can also be applied to treatment of chronic liver disease [22]. Some studies have reported that leptin administration can correct many of the metabolic syndromes of liver steatosis [13,21,23]. This may be because leptin is one of the key regulators of inflammation in chronic liver steatosis [24]. Although some have also reported that leptin resistance plays an important role in liver steatosis [14], this did not correlate with our findings of chronic liver steatosis programming. In our study, we also showed that decreased leptin expression levels in mRNA and proteins, leptin receptor mRNA in the DEX group, and leptin methylation are involved in the process of chronic liver steatosis and are important disease-causing factors.

Melatonin is a serotonin-derived neurohormone formed primarily in the brain by the pineal gland of all mammals [25]. Melatonin concentrations were elevated at night and were low during the day [6,26], and chronic phase shifts were found in maternal and fetal hormonal levels [3,6,27]. In terms of programming, maternal melatonin secretion plays a role in programming the energy metabolism of the offspring [3,27]. Melatonin can attenuate plasma liver enzymes among patients with liver steatosis [28], thereby lessening liver damage [26]. Leptin plasma levels have previously been found to be significantly elevated in patients with non-alcoholic steatohepatitis after melatonin treatment [28]. In our study, we demonstrated that the administration of melatonin beginning at pregnancy can reverse leptin methylation and its expression in prenatal dexamethasone-exposed adult offspring with chronic liver steatosis. Thus, melatonin plays a protective role in chronic prenatal stress-induced liver steatosis and fatty liver development.

Liver epigenetic phenotype predetermines individual susceptibility to liver steatosis in mice [2,9]. Leptin methylation pattern can be influenced by diet-induced obesity for 11 weeks [29]. Our study also found that the methylation status of the leptin gene in these animals could be maintained until postnatal day 120. It was rescued by the administration of melatonin for DNMT and HDAC, associated with fatty liver disease [2]. Distinctive genomic methylation patterns ensure the inactivity of specific promoters during development [3,10]. Loss of genomic and repetitive sequences of cytosine methylation is accompanied by increased levels of repeat-associated transcripts and maintains DNMT proteins in the liver [9]. Our study confirmed that liver DNMT activity was increased in prenatal dexamethasone and decreased after melatonin administration. We also found that prenatal dexamethasone exposure increased liver HDAC activity; however, melatonin did not reduce it in the 120 days of the experiment [2]. Further study is needed to provide insights into the role of the different levels of HDAC and DNMT expression in melatonin-treated chronic liver steatosis.

In nutrient-sensing pathways, evidence suggests that sirtuins play important roles in regulating fatty liver disease-related metabolic processes [30]. Our previous publication showed there was no significant change in liver SIRT1 after prenatal high fat, but it significantly deceased in maternal and post-weaning high fat diet [31]. There was no paper mentioned about whether SIRT1 was involved in the prenatal dexamethasone liver steatosis model, and this needs further study to prove. It was reported that the expression of SIRT1 and SIRT2 in metabolic syndrome rats was reduced in white adipose tissue at six months old [32]. Our unpublished data showed that the retroperitoneal fat SIRT1 was not altered in the prenatal dexamethasone given at postnatal 120 days. The mechanism and the relations of this important pathway in adipose tissue and liver steatosis need further study to clarify it.

The response to noxious insults is typically deregulated, prolonged, and inflammatory in nature [10,33]. TNF-α and IL-6 activate specific intracellular pathways in hepatocytes [34], and are critical inflammatory mediators involved in many diseases [10,35]. Kupffer cells are major producers of TNF-α and IL-6 levels and they modulate their activities [34]. Therefore, in the DEX group with higher TNF-α and IL-6 expression levels, this suggests that steatosis may be related to Kupffer cell dysfunction or activation [34,36], TGF-β signaling increased in fatty livers with inflammation [37,38]. In this study, liver inflammation with higher IL-6, TNF-α, and TGF-β expression was observed in the DEX group. Some studies have previously reported that apoptosis is the main process contributing to disease progression in NAFLD [39]. However, in this study, on the chronic stage of prenatal steroid administration**,** we did not find any indications of increased apoptosis in the DEX group. The mechanism of programming chronic liver steatosis was found to be dependent on inflammation, but not on cellular apoptosis, and melatonin was found to reverse it. Thus, it is more important to develop a strategy to rescue chronic liver steatosis via anti-inflammatory rather than anti-apoptotic effects.

## 4. Materials and Methods

### 4.1. Animals

Sprague-Dawley (SD) rats were housed in the animal care facility of Chang Gung Memorial Hospital, Kaohsiung, Taiwan in a 12-h light/dark cycle with the lights on at 07:00. [2]. Pregnant rats were checked for litters daily at 10:00. Sprague-Dawley female rats were allowed to mate with male rats for 24 h. One day later, female rats were separated from the male rats and housed individually in a standard plastic home cage. After confirmation of pregnancy on the 14th day after mating, pregnant females were either randomly divided for the prenatal steroid exposure paradigm or left undisturbed until delivery. The day of birth was designated as postnatal day 0 (PND 0). Rat pups were weaned at PND 21 and had access to standard chow and water *ad libitum*. Only male rats were used in this study. The care and use of the laboratory animals strictly followed the protocol of the Institutional Animal Care and Use Committee, which was approved by the Animal Ethics Committee of the Chang Gung University, Kaohsiung, Taiwan (Permit Number: 2013060701, 19 July 2013). Six rats were used in each group and a total of 24 animals were used in this study. The condition of the animals was monitored twice a day. The rats were sacrificed with ketamine to minimize the suffering of the animals when signs of infection or respiratory or gastrointestinal symptoms appeared. The rats were anesthetized with an intramuscular injection of ketamine (10 mg/kg; Pfizer, Taipei, Taiwan) for termination. Immediately after the termination of the rats, the liver tissues were taken and weighted together with intracardiac blood samples and placed into EDTA-containing vials. Assays to determine the triglyceride and cholesterol levels and aspartate transaminase (AST) and alanine transaminase (ALT) activity were carried out using a standard autoanalyzer (Hitachi model 7450, Tokyo, Japan).

### 4.2. Prenatal Dexamethasone Exposure Paradigm

Pregnant Sprague-Dawley rats at gestational day 14–21 were intraperitoneally administered dexamethasone (0.1 mg/kg/day) [2,40]. The chronic effects of prenatal programming by glucocorticoid were assessed at postnatal day 120.

### 4.3. Melatonin Treatment 

Melatonin was used clinically in oral form. We administered a new group with melatonin dissolved in water. Rats drank about 25 mL/day and the average daily intake of melatonin was estimated to be 1 mg/kg/day in pregnant rats from gestational day 14–21 to postnatal day ~120. Melatonin was prepared three times a week by dissolving the melatonin (16 mg) in ethanol (1 mL, 100% *v/v*). This solution was diluted with distilled water to a final concentration of 40 mg/L. The bottles were covered with aluminum foil to protect them from light exposure [2].

### 4.4. Localization of Oil Red Stain and Analysis 

To study the expression of liver lipid proteins, we cut 2–3-μm thick sections of the frozen liver tissue and mounted it on coating slides. Tissue sections were incubated with 3% hydrogen peroxide for 10 min to block endogenous peroxidase activity. The sections were stained with Oil Red O. The number of positively-stained cells was counted from a total of five hundred hepatocytes in each group [2].

### 4.5. RNA Isolation and Real-Time PCR 

To quantitate the amount of RNA in tissue, we used real-time PCR (RT-PCR) with the LightCycler^®^ 480 Real-time PCR system (Roche Co., Mannheim, Germany). Total RNA was extracted from the liver tissue using Trizol reagent (Invitrogen; Boston, MA, USA). RNA was quantified by A260 and its integrity was verified by agarose gel electrophoresis using ethidium bromide for visualization. For RT-PCR, the reagent mixture was prepared according to the protocol provided by the manufacturer (Protech Technology, Taipei, Taiwan). Two micrograms of total RNA were used to generate cDNA using an oligodeoxynucleotide primer (oligo dT15) following the protocol for transcription (Promega, Madison, WI, USA). PCR was performed in 20 μL LightCycler^®^ 480 SYBR Green I Master (Roche Co., Mannheim, Germany) containing 10 nmol forward primers and reverse primers, and approximately 10 ng cDNA. The primers sequences were as follows: the leptin primers were 5′-TCTCCGAGACCTCCTCCATCT-3′ for the forward primer and 5′-TTCCAGGACGCCATCCAG-3′ for the reverse primer. The β-actin primers were 5′-TCACCCACACTGTGCCCATCTACGA-3′ and 3′-GGTAACCGTTACTCGCCAAGGCGAC-5′, respectively. Amplification and detection was performed with the LightCycler^®^ 480 Real-time PCR system [2]. The validation experiments were done in triplicate and the amplification efficiencies were validated. RNA expression levels were normalized to β-actin RNA levels and calculated according to the ΔΔCt method [2].

### 4.6. Western Blotting Analysis 

After treatment, the tissues were dissected from samples and frozen immediately in liquid N_2_. The tissue was homogenized in a buffer and centrifuged at 14,000× *g*. Proteins (40 µg) from the supernatant of each sample were separated by SDS-PAGE and transferred to polyvinylidene difluoride membranes by electrophoresis. The membranes were blocked in TBST buffer containing 5% non-fat milk powder for 1 h at room temperature. Immunoblotting assays were performed using specific primary antibodies: primary monoclonal leptin (1:1000; Abcam, ab16227, Cambridge, MA, USA), and tumor necrosis factor alpha (TNF-α) antibody (1:1000; Cell Signaling, #3707, Danvers, MA, USA), followed by a secondary alkaline phosphatase-conjugated anti-IgG antibody (1:5000; Promega, Madison, WI, USA). The Western blots were visualized using the Blot AP System (Promega) [2,41,42].

### 4.7. Histone Extraction 

Histones were extracted (EpiSeeker Histone Extraction Kit, Abcam, ab113476, Cambridge, MA, USA) from 100 mg tissue, which was then cut into small pieces (1–2 mm^3^) with scissors. The tissue pieces were then transferred to a Dounce homogenizer and homogenized with 1X pre-lysis buffer at 200 mg/mL. The total mixture volume was transferred to a 2 mL vial and centrifuged at 10,000 rpm for 1 min at 4 °C, then the supernatant was removed. The pellet was resuspended in lysis buffer to incubate on ice for 30 min. Then, it was centrifuged at 12,000 rpm for 5 min at 4 °C and discarded the pellet. The supernatant was transferred into 0.3 volumes of the balance-DTT buffer and quantified the protein concentration [2].

### 4.8. HDAC/DNMT Activity Assay 

The measurement of histone deacetylase (HDAC) and DNA methyltransferase (DNMT) activity was performed using an EpiQuiktm HDAC, DNMT activity/inhibition assay kit (ET Epigentek, Farmingdale, NY, USA) according to the manufacturer’s instructions. For the determination of HDAC and DNMT activity, 100 μg nuclear extracts from SD rat liver tissue at 6 μg nuclear protein concentrations were added to each strip, which contained stably captured antibody substrate. Samples were incubated at 37 °C for 60 min to allow each activity assay to bind to the enzyme substrate. Subsequently, the high affinity acetylated histone antibody (1 mg/mL) was used to recognize un-deacetylated substrate. The amount of un-deacetylated substrate was inversely proportional to the enzyme activity. Finally, the enzymatic activity of HDAC and DNMT was detected using a micro plate reader at 450 nm following an ELISA-like reaction. HDAC and DNMT activity was expressed as relative optical density values per hour per mg of protein sample (OD/h/mg) [2,43].

### 4.9. Bisulfite Modification 

Bisulfite modification was performed based on the principle that bisulfite converted unmethylated cytosine residues into uracil, whereas methylated cytosine residues remained unaffected. After bisulfite conversion, methylated and unmethylated cytosines can be determined by different methods such as methylation specific PCR (MSP) and direct sequencing. Bisulfite treatment of DNA was performed using a Zymo methylation Gold kit (Zymo Research, CA, USA) according to the manufacturer’s instructions [2].

### 4.10. Methylation Specific qPCR 

The MSP was assessed using nested PCR with 2 μg of bisulfite-treated DNA in the first round of the PCR. One microliter (1 μL) of bisulfite-treated DNA was used for two separate nested PCR with 10 μM of each primer specific for methylated and unmethylated sequences as follows: the primers used were specific for bisulfite-converted DNA methylated or unmethylated sequences. The leptin methylated primers were 5′-GTTTAGTAGTTGTTGGTCGGATTTC-3 for the forward primer and 5-CAACCTAATACTCCATTCTAAACGC-3 for the reverse primer. The leptin unmethylated primers were 5-TTTAGTAGTTGTTGGTTGGATTTTG-3 and 5-AACCTAATACTCCATTCTAAACACC-3, respectively. Amplification, detection, and post-amplification were performed according to the protocol of the LightCycler 480 Real-time PCR system [2].

### 4.11. TdT-Mediated dUTP Biotin Nick End Labeling (TUNEL)

Liver cellular apoptosis expression was studied. TUNEL expression was detected using a ApopTag^®^ Plus Peroxidase In Situ Apoptosis Detection Kit (CHEMICON International Inc., Temecula, CA, USA) according to the manufacturer’s protocol [2,42]. Deparaffinized sections were washed with distilled water and treated with Protein Digestion Enzyme for 15 min at 37 °C. The number of positively-stained cells was counted from a total of five hundred hepatocytes in each rat.

### 4.12. Cytokine Secretion with Enzyme-Linked Immunosorbent Assay (ELISA)

The cytokine levels in the plasma were analyzed using interleukin 6 (IL-6) and transforming growth factor beta (TGF-β) commercial ELISA kits (R & D Systems, Minneapolis, MN, USA) according to the manufacturer’s protocol. A standard curve for using recombinant cytokine was generated for each assay.

### 4.13. Immunohistochemistry Stain

Formalin-fixed, well-preserved SD rat tissue blocks from surgically resected liver specimens were used for immunohistochemical study, as described in our previous report [2]. The primary antibody used in this experiment was TNF-α. The slides were washed with PBS and incubated with MACH4 universal HRP polymer kit (BioCare M4U534, Pacheco, CA, USA) for 15 min. After washing with PBS, a chromogenic reaction was developed by incubating with Betazoid DAB chromogen kit (BioCare BDB2004, CA, USA).

### 4.14. Statistical Analysis

SPSS for Windows 13.0 version was used for the statistical analysis. Continuous variables were analyzed using independent *t*-tests or analysis of variance (ANOVA). The data were presented as the mean ± standard error (SE). *p* < 0.05 was considered to be statistically significant.

## 5. Conclusions

In summary, our study showed that programming of chronic liver steatosis in adult offspring was induced by prenatal exposure to dexamethasone. One major mechanism of this was increased leptin methylation and decreased leptin expression. Melatonin may attenuate chronic liver steatosis by reducing leptin methylation and decreasing liver inflammation; however, this does not occur via cellular apoptosis or the HDAC pathway.

## Figures and Tables

**Table 1:** Weight and laboratory data (SEM).

	Vehicle	DEX	DEX+MEL
body weight (gm)	527.08 ± 8.77	521.50 ± 9.93	479.56 ± 15.86 *
liver weight (gm)	16.98 ± 0.77 *	17.94 ± 0.87	16.96 ± 0.80 *
liver/BW (%)	3.19 ± 0.11	3.49 ± 0.12	3.53 ± 0.10
triglyceride (mg/dL)	74.85 ± 2.15	74.78 ± 3.39	64.13 ± 3.54 *
cholesterol (mg/dL)	123.15 ± 9.85	126.33 ± 11.78	84.25 ± 5.89 * ^†^
AST (U/L)	81.0 ± 5.13	89.44 ± 7.57	87.50 ± 6.03
ALT (U/L)	44.85 ± 2.69	47.63 ± 5.36	48.25 ± 5.89

DEX: prenatal dexamethasone; DEX+MEL: melatonin treatment after prenatal dexamethasone; SEM: standard error of means; AST: aspartate transaminase; ALT: alanine transaminase; BW: body weight. * *p* < 0.05 compared with DEX group; ^†^ *p* < 0.05 compared with vehicle group.

## Abbreviations

ALT	alanine transaminase
AST	aspartate transaminase
DEX	dexamethasone
DNMT	DNA methyltransferase
HDAC	histone deacetylases
MEL	melatonin
MSP/USP	methylation-specific PCR/unmethylation-specific PCR
NAFLD	steatosis in non-alcoholic fatty liver disease
TGF-β	transforming growth factor beta
TNF-α	tumor necrosis factor alpha
